# Evaluation of maternal and neonatal complications in preeclamptic twin versus singleton pregnancies: a retrospective study

**DOI:** 10.1007/s00404-025-08068-6

**Published:** 2025-06-06

**Authors:** Javier Sánchez-Romero, María Rodríguez-Contreras, Valeria Rolle, Romina Sol Liandro, Miriam Pertegal-Ruiz, María Muñoz-Contreras, José Eliseo Blanco-Carnero, Catalina De Paco Matallana

**Affiliations:** 1 Department of Obstetrics and Gynecology. ‘Virgen de La Arrixaca, University Hospital, 30120 Murcia, Spain; 2 Department of Obstetrics, Gynecology, Surgery and Pediatrics, University of Murcia, 30120 Murcia, Spain; 3 Biostatistics and Epidemiology Platform at Instituto de Investigación Sanitaria del Principado de Asturias, Oviedo, Asturias Spain

**Keywords:** Preeclampsia, Twin pregnancy, Singleton pregnancy, Maternal complications, Neonatal complications

## Abstract

**Purpose:**

This study aims to compare maternal and neonatal outcomes in singleton and twin pregnancies complicated by preeclampsia, emphasizing differences between preterm and term deliveries.

**Methods:**

This is a retrospective study conducted at "Virgen de la Arrixaca" University Hospital (Murcia, Spain), from 2009 to 2020. Maternal demographic data and maternal and neonatal outcomes were collected from hospital records. Pregnancies were stratified by delivery before and after 37 weeks of gestation.

**Results:**

The study included 161 singleton pregnancies and 77 twin pregnancies, all complicated by preeclampsia. Preterm delivery rates (< 37 weeks) were significantly higher in twin compared to singletons (79.2% vs. 48.4%). The mean maternal hospital stay was longer in twins (9.0 days) than for singletons (7.6 days). Maternal complications occurred in 13.7% of singleton pregnancies and 28.6% of twin pregnancies (*p* = 0.006), with maternal hemorrhage more frequent in twins (22.1% vs. 9.3%; *p* = 0.007). Maternal complications were more common in twin pregnancies (OR = 3.13; 95%CI 1.38–7.10). Cesarean delivery (OR = 2.00; 95%CI 0.85–4.66) and BMI (OR = 0.96; 95%CI 0.90–1.03) were not associated with the maternal composite outcome.

Neonatal complications occurred in 29.2% of singleton pregnancies and 30.0% of first twin and 27.3% of second twin (*p* = 0.890 and 0.790 respectively). Factors associated with neonatal complications included birthweight (OR 0.99; 95%CI 0.99–0.99) and delivery between 34 and 37 weeks of gestation (OR = 0.08; 95%CI 0.01–0.59) and delivery after 37 weeks of gestation (OR = 0.04; 95%CI 0.01–0.46).

**Conclusions:**

Maternal complications were more frequent in twin pregnancies complicated by preeclampsia, while neoantal complications were more likely to occur in cases of preterm preeclampsia.

## What does this study add to the clinical work

**Table:** 

Maternal complications in preeclampsia were linked to twin pregnancies, while neonatal complications were more frequent in preterm preeclampsia.	
This study highlights no significant differences in neonatal outcomes between singleton and twin pregnancies with preeclampsia, underscoring the impact of delivery timing on complications.	

## Introduction

Preeclampsia (PE) is a severe, pregnancy-specific hypertensive disorder affecting 2% to 8% of pregnancies, with a higher prevalence and earlier onset in twin pregnancies [1]. The increasing use of assisted reproductive technologies has raised twin pregnancy rates to 3–4% in developed countries [2, 3], further amplifying the incidence and severity of PE and its associated complications [1, 4]. Twin pregnancies also carry additional risk factors, such as obesity and gestational diabetes, which further increase the susceptibility to PE [5].

PE is a multisystem progressive disorder characterized by new-onset hypertension and proteinuria occurring after 20 weeks of gestation [6]. Among its forms, preterm PE poses a higher risk of complications compared to term PE. However, globally, term PE contributes more significantly to morbidity due to its higher incidence [7, 8].

PE is associated with various complications, including placental abruption, HELLP syndrome, eclampsia, pulmonary edema, renal failure, and neurological deficits [9]. Additionally, it increases neonatal morbidity and mortality, as it is often associated with fetal growth restriction and preterm birth [7, 8, 10]. In twin pregnancies complicated by PE, the likelihood of prolonged neonatal intensive care admission is higher [11]. Prematurity related to PE can result in neonatal complications such as necrotizing enterocolitis, bronchopulmonary dysplasia, and intraventricular hemorrhage [7].

In twin pregnancies, the incidence of preeclampsia (PE) is approximately 9%, which is three times greater than in singleton pregnancies [12]. However, twins are typically delivered at an earlier gestational age than singletons. As a result, comparing the overall PE rates between twin and singleton pregnancies underrepresents the true relative risk of preterm PE in twins, which is nine times higher [12]. However, there is limited research comparing the clinical characteristics of preeclampsia and maternal and neonatal outcomes in twin versus singleton pregnancies [11, 13]. A notable multicenter study conducted by Sibai et al. in the early 2000s examined neonatal outcomes in singleton versus twin pregnancies complicated by PE [14]. However, neonatal management has evolved significantly over the last two decades, leaving the contemporary neonatal consequences of PE-related complications less well understood [4, 15].

This study aimed to compare the clinical features of preterm and term PE in singleton versus twin pregnancies and to evaluate maternal and neonatal complications associated with PE.

## Methods

This retrospective cohort study included all patients diagnosed with preeclampsia (PE) from 2009 to 2020 at ‘Virgen de la Arrixaca’ Clinic University Hospital (Murcia, Spain). Data were extracted from clinical records. The study was conducted in accordance with the Declaration of Helsinki, and the protocol received approval from the local Institutional Review Board (CEID 981/2024).

Clinical features, obstetric and delivery management, as well as maternal and neonatal outcomes, were obtained from clinical records. Gestational age and chorionicity were confirmed via first-trimester ultrasonography. PE was defined based on criteria from the American College of Obstetricians and Gynecologists (ACOG) [9], as chronic or gestational hypertension (systolic blood pressure ≥ 140 mmHg and/or diastolic blood pressure ≥ 90 mmHg, on at least two occasions, 4 h apart, and developing after 20 weeks of gestation in a previously normotensive woman), along with at least one of the following: proteinuria (≥ 300 mg/24 h, protein-to-creatinine ratio ≥ 30 mg/mmol or urinary dipstick testing ≥ 2 +), renal insufficiency with serum creatinine > 97 µmol/L in the absence of underlying renal disease, hepatic dysfunction with blood concentration of transaminases more than twice the upper limit of normal (≥ 65 IU/L for our laboratory), thrombocytopenia (platelet count < 100 000/µL), neurological complications (for example, cerebral or visual symptoms) or pulmonary edema. PE was also classified as preterm and at term PE base on FIGO guideline [5].

Management of PE follows standardized local clinical guidelines. Antihypertensive treatment targets blood pressure below 150/100 mmHg, using oral labetalol as the first-line agent. Intravenous administration or additional drugs such as labetalol or hydralazine are reserved for severe cases. Magnesium sulfate is indicated for seizure prophylaxis in patients with severe features or eclampsia. Fetal monitoring includes serial ultrasound assessments of growth, amniotic fluid, and Doppler studies, as well as regular cardiotocography starting from viability. Pregnancy is electively terminated at 37 weeks in cases without severe features and from 34 weeks in severe cases, or earlier if maternal or fetal conditions warrant immediate delivery.

Newborns were evaluated immediately after delivery by a neonatologist in twin pregnancies and in singleton pregnancies requiring operative delivery or cesarean section. Neonatal composite outcome was considered when newborn required CPAP, invasive mechanical ventilation, when a respiratory distress syndrome, intraventricular hemorrhage, necrotizing enterocolitis, sepsis or retinopathy was diagnosed or if newborn developed anemia that required blood transfusion.

Maternal composite outcome was considered when a HELLP syndrome, eclampsia, placenta abruption, postpartum hemorrhage, sepsis or thrombosis was diagnosed.

### Statistical analysis

Prior to analysis, all variables were checked for normality and homogeneity of variance using the Kolmogorov–Smirnov and Levene tests, respectively. Categorical variables were compared using the chi-square test. To evaluate the existence of significant differences between groups (singleton vs. twin pregnancy), a Student's t-test or ANOVA was performed for unpaired samples if normality was met, while a Mann–Whitney U or Kruskal–Wallis test was used for non-normal distributions. When multiple comparisons were required, Bonferroni correction was applied. Data are presented as mean (standard deviation) for continuous variables and absolute frequency (relative frequency) for categorical variables. When continuous variables did not follow a normal distribution, they are reported as median (interquartile range).

To assess the relationships between potential confounders (nulliparity, gestational age at delivery, conception method, mode of delivery, and BMI) and maternal or neonatal complications, logistic regression models were built for each parameter. Variables included in the models where those identified as candidates (*p* < 0.2 in the univariate models), as well as potential confounders identified in previous reports. Potential confounders were retained in the final model if there was a ≥ 10% change between the crude and adjusted odds ratios (OR). Model goodness-of-fit was assessed using the Hosmer–Lemeshow test. For the neonatal outcome, separate regression models were performed for singleton and twin pregnancies. In the model including twins, each neonate was treated as an individual observation; however, robust standard errors were estimated using the mother’s unique identifier as a clustering variable, to account for the correlation between siblings from the same pregnancy. Gestational age at delivery was included as a covariate since no complications were observed in the second twin beyond 37 weeks of gestation. The reported results include odds ratios (OR) or estimates, along with their corresponding 95% confidence intervals (CI) and p-values. The significance threshold was adjusted using the False Discovery Rate (FDR) method. Model assumptions were visually assessed.

A *p*-value < 0.05 was considered statistically significant. All statistical analyses were performed using R statistical software (version 4.3.0, R Core Team, 2023) and Stata/BE 18.0 (StataCorp, College Station, Texas, USA).

## Results

### Maternal-neonatal characteristics and clinical outcomes

238 pregnant women with preeclampsia were recruited. Of them, 161 (67.6%) were singleton and 77 (32.4%) twin pregnancies. 78 (48.4%) singleton and 61 (79.2%) twin pregnancies with PE required delivery before 37 weeks. Baseline maternal characteristics are shown in Table 1. Maternal age and *assisted reproductive techniques* (ART) conception rates were higher in twin pregnancies, whereas higher BMI, diabetes mellitus, and induced labor were more likely in singleton pregnancies.

**Table 1:** Baseline maternal characteristics. Values are means (standard deviation) or absolute frequency (relative frequency)

Variable	Singleton (N = 161)	Twin (N = 77)	*p*
Maternal age (years)	33.2 (5.82)	35.6 (5.81)	**0.016**
BMI (kg/m^2^)	27.8 (6.44)	25.6 (5.0)	**0.003**
Ethnicity			
Black	5 (3.1%)	3 (3.9%)	0.620
Oriental	1 (0.6%)	1 (1.3%)	
Caucasian	155 (96.3%)	73 (94.8%)	
Smoke	19 (11.8%)	8 (10.4%)	0.830
Conception			
Spontaneous	148 (91.9%)	35 (45.5%)	** < 0.001**
ART	13 (8.1%)	42 (54.5%)	
Nulliparity	93 (57.8%)	48 (62.3%)	0.573
Chronic Hypertension	22 (13.7%)	5 (6.5%)	0.123
Diabetes Mellitus			
Type 1	2 (1.2%)	0 (0%)	** < 0.001**
Type 2	23 (14.3%)	1 (1.3%)	
APL Syndrome	2 (1.2%)	0(0%)	1
Gestational age at PE onset			
Preterm PE	78 (48.5%)	61 (79.2%)	** < 0.001**
At term PE	83 (51.6%)	16 (20.7%)	
Gestational age at delivery (weeks)*	37.0 (3.9)	35.9 (2.1)	**0.001**
< 28 weeks	6 (3.7%)	1 (1.3%)	** < 0.001**
28–32 weeks	24 (14.9%)	4 (5.2%)	
32–34 weeks	9 (5.6%)	9 (11.7%)	
34–37 weeks	43 (26.7%)	53 (68.8%)	
> 37 weeks	79 (49.1%)	10 (13.0%)	
Labor onset			
Spontaneous	28 (17.4%)	13 (16.9%)	**0.032**
Induced	96 (59.6%)	34 (44.2%)	
No labor onset	37 (23.0%)	30 (39.0%)	
Type of delivery			
Vaginal	57 (35.4%)	26 (33.8%)	0.885
Cesarean section	104 (64.6%)	51 (66.2%)	
Steroids	43 (26.7%)	29 (37.7%)	0.085
Gestational age at steroids administration, weeks*	31.6 (5.4)	33.0 (3.7)	0.207

Statistically significant comparisons are represented in bold

*BMI* Body Mass Index, *ART* Assisted Reproductive Techniques, *APL* Antiphospholipid syndrome. * Variables reported as median (Interquartile range)

### Neonatal complications

Table 2 shows the fetal and neonatal baseline outcomes. Neonatal complications were observed in 47 singletons (29.2%), 23 in first twin (30.0%; *p* = 0.890) and in 21 s twin (27.3%; *p* = 0.790) babies. An increased neonatal intensive care unit (nICU) admission rate and neonatal complications were higher in singleton with preterm preeclampsia (p = 0.001 and *p* = 0.012, respectively).

**Table 2:** Fetal and neonatal outcomes

Variable	Singleton (*N* = 161)	First twin (*N* = 77)	Second twin (*N* = 77)	*p*
Outcome				
Livebirth	156 (96.9%)	75 (97.4%)	77 (100%)	0.728; 0.295*
Neonatal Death	1 (0.6%)	1 (1.3%)	0	
Stillbirth	4 (2.5%)	1 (1.3%)	0	
Arterial cord blood pH	7.30 (0.12)	7.31 (0.07)	7.29 (0.11)	0.940; 0.687*
Arterial cord blood pH < 7	0	1 (0.01%)	0	0.106; 1*
Venous cord blood pH	7.33 (0.07)	7.35 (0.05)	7.35 (0.03)	0.096; 0.436*
Apgar test score 5`	10 (1)	10 (0)	10 (1)	0.069; 0.294*
Birthweight, grams **	2,317.2 (445.8)	2,160.4 (480.7)	2,538.4 (881.4)	0.171; 0.026*
Birthweight, centile	30 (58)	30 (50.5)	15 (33)	0.999; 0.005*
Birthweight < 10th centile	48 (30.6%)	19 (25%)	32 (41.6%)	0.379; 0.097*
nICU admission	28 (17.8%)	6 (7.8%)	10 (13.0%)	**0.041**; 0.345*
nICU admission time, days	15.0 (19)	7.5 (5)	7.0 (5)	0.174; 0.029*
Neonatal complications	47 (29.2%)	23 (30.0%)	21 (27.3%)	0.890; 0.790
Ventilation	36 (22.4%)	13 (16.9%)	8 (10.4%)	0.300; 0.024*
Intubation	16 (9.9%)	2 (2.6%)	2 (2.6%)	0.060; 0.058*
Intubation time, days	2.0 (2)	1 (66)	3.5 (1)	0.995; 0.659*
CPAP	30 (18.6%)	8 (10.4%)	6 (7.8%)	0.099; 0.029*
CPAP time, days	0 (1)	2 (4)	1.75 (2)	0.001; 0.012*
RDS	36 (22.4%)	12 (15.6%)	7 (9.1%)	0.204; 0.013*
Phototherapy	29 (18.0%)	12 (15.6%)	16 (20.8%)	0.602; 0.674
Phototherapy, days	2 (2)	1 (1)	1.5 (1)	0.389; 0.789*
Intraventricular hemorrhage	2 (1.2%)	2 (2.6%)	0	0.464; < 0.001*
Anemia	7 (4.3%)	4 (5.2%)	2 (2.6%)	0.790; 0.492*
Blood transfusion	4 (2.5%)	2 (2.6%)	2 (2.6%)	0.976; 0.982*
Necrotizing enterocolitis	4 (2.5%)	1 (1.3%)	0	0.550; < 0.001*
Retinopathy	6 (3.7%)	1 (1.3%)	0	0.316; < 0.001*
Sepsis	5 (3.1%)	2 (2.6%)	3 (3.9%)	0.812; 0.779*

Values are median (Interquartile range) or absolute frequency (relative frequency). Statistically significant comparisons are represented in bold

*nICU* neonatal intensive care unit, *IMV* Invasive Mechanical Ventilation. *RDS* Respiratory Distress Syndrome. *Comparing second twin with singleton with Bonferroni correction. **Variables reported as means (standard deviation)

**Fig. 1:**
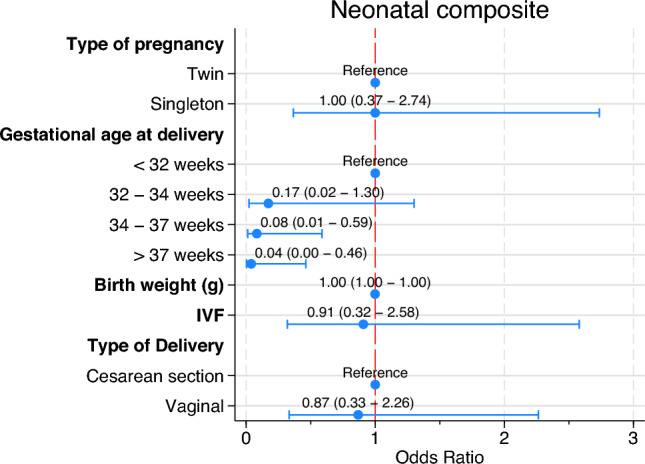
Multivariate logistic regression model for neonatal complications

A logistic regression model for fetal and neonatal complications is shown in Fig. 1. Lower birthweight (OR 0.99; 95%CI 0.99–0.99; p < 0.001), delivery between 34 and 37 weeks (OR = 0.08; 95%CI 0.01–0.59; *p* = 0.013) and after 37 weeks (OR = 0.04; 95%CI 0.01–0.46; *p* = 0.010) were the variables associated with neonatal complications. No differences were observed between singleton and twin pregnancies (*p* = 0.999) or mode of delivery (*p* = 0.716).

### Maternal complications

Table 3 shows the maternal outcomes. No maternal death was reported. Maternal complications were observed in 22 (13.7%) singleton and in 22 (28.6%) twin pregnancies (*p* = 0.011). Maternal composite outcome was observed in 8 twin pregnancies (50%) complicated by at term PE. Admission time was longer in twin pregnancies with at term preeclampsia (*p* < 0.001).

**Table 3:** Maternal outcomes

Variable	Singleton (*N* = 161)	Twin (*N* = 77)	*p*
Admission time, days*	6.0 (6.0)	7.0 (4.0)	**0.011**
Maternal complications	22 (13.7%)	22 (28.6%)	**0.006**
Preterm PE	16 (20.5%)	14 (23.0%)	0.729
At term PE	6 (7.2%)	8 (50.0%)	** < 0.001**
HELLP syndrome	3 (1.9%)	2 (2.6%)	0.712
Eclampsia	3 (1.9%)	1 (1.3%)	0.751
Abruptio placentae	3 (1.9%)	0	0.553
Postpartum hemorrhage	15 (9.3%)	17 (22.1%)	**0.007**
Blood transfusion	10 (6.2%)	9 (11.7%)	0.145
Uterine tamponade	1 (0.6%)	4 (5.2%)	0.021
Thrombosis	1 (0.6%)	0	1
Maternal death	0	0	

Values are means (standard deviation) or absolute frequency (relative frequency). Statistically significant comparisons are represented in bold

*PE* Preeclampsia. *Variables reported as median (Interquartile range)

**Fig. 2:**
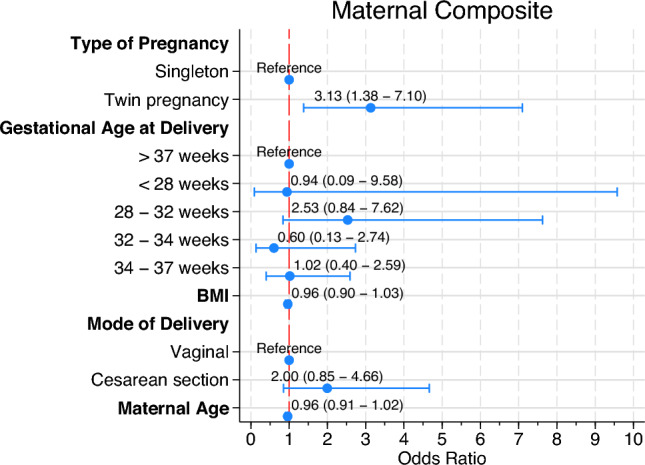
Multivariate logistic regression model for maternal complications

Figure 2 presents a logistic regression model for maternal complications. Twin pregnancy (OR = 3.13; 95%CI 1.38–7.10; p = 0.006) was the only variable associated with maternal composite outcome. No differences were observed in BMI (OR = 0.96; 95%CI 0.90–1.03; *p* = 0.297) or cesarean delivery (OR = 2.00; 95%CI 0.85–4.66; *p* = 0.110).

## Discussion

This study contributes to existing literature by analyzing both maternal and neonatal outcomes in singleton and twin pregnancies affected by preeclampsia. Maternal complications were more likely in twin pregnancies. Furthermore, neonatal complications were predominantly associated with preterm preeclampsia. The mode of delivery had no effect on maternal or neonatal outcome.

A key finding of our study is that neonatal outcomes did not significantly differ between singleton and twin pregnancies when matched for gestational age at delivery. This suggests that whether the pregnancy is singleton or twin does not independently influence fetal outcomes, but rather that gestational age is the primary determinant of neonatal outcome.

Although previous studies have not directly compared neonatal outcomes between singleton and twin pregnancies after adjusting for gestational age, Connolly et al. reported no significant differences in neonatal mortality or nICU admission among pregnancies complicated by preeclampsia [15]. In contrast, Sibai et al. found an increased risk of nICU admission in twin pregnancies (RR = 2.24; 95% CI: 1.47–3.40) [14].

We reported a stillbirth rate of 2.5% and 1.3% in singleton and twin respectively, and neonatal death rate of 0.6% and 1.3%, in preterm preeclampsia. These outcomes are consistent with those reported by previous studies [13, 15–18].

In our cohort, neonatal complications and nICU admissions were more common in singleton pregnancies with preterm preeclampsia (*p* = 0.012 for both outcomes). This finding appears to contrast with data from Connolly et al. and Weiner et al., who reported nICU admission rates of 80% and 40.7% for singleton pregnancies, and 88% and 48.5% for twin pregnancies, respectively [11, 15]. These differences were not statistically significant (*p* = 0.16 and *p* = 0.14, respectively). Such discrepancies may be attributed to variations in study populations and neonatal care protocols. Additionally, both studies began recruitment in 2009, several years prior to the publication of the ASPRE trial [19], probably because of that they found an increased early-onset preeclampsia with poorer neonatal outcomes.

The multivariate regression model (Fig. 1) identified low birthweight and delivery after 34 weeks as the variables significantly associated with neonatal complications. Interestingly, cesarean delivery showed no significant association with neonatal complications. No prior studies have employed multivariate analysis to examine the role of singleton versus twin pregnancies in both preterm and term preeclampsia. Although cesarean delivery has historically been associated with neonatal respiratory morbidity [20], recent evidence does not support this relationship [21].

Maternal complications were observed in 22 (13.7%) singleton and in 22 (28.6%) twin pregnancies (*p* = 0.011). Postpartum hemorrhage was the most frequent maternal complication. It was notably more common in twin pregnancies with term preeclampsia. In contrast, Ni et al. reported a lower incidence of postpartum hemorrhage [16], which aligns with the general observation that postpartum hemorrhage is less frequent in pregnancies unaffected by preeclampsia [22]. Our findings revealed a higher incidence of HELLP syndrome (1.9% and 2.6%, respectively) compared to previous studies [4, 13, 15, 16]. The incidence of eclampsia in our study (1.9% in singleton and 1.3% in twin pregnancies) was slightly higher than that reported in previous studies investigating maternal complications in pregnancies affected by PE (0.6–2.0%) [4, 13, 15, 16]. However, our findings are consistent with the 2.6% eclampsia rate observed in large observational cohorts of singleton pregnancies [23]. Multivariate regression model (Fig. 2) for maternal complications revealed an increased risk in twin pregnancies. The mode of delivery appears to have no impact on maternal outcome. Previous studies reported an increased risk of maternal complications in pregnancies complicated by preeclampsia[24], and an increased maternal morbidity in cesarean section [25]. Although, cesarean delivery seems to increase maternal complications in our regression model, it did not reach statistical significance (*p* = 0.110).

Our findings highlight the increased risk of maternal complications, particularly postpartum hemorrhage, in twin pregnancies complicated by preeclampsia. This is consistent with the established understanding that multiple gestation is an independent risk factor for postpartum hemorrhage, which may be further exacerbated by preeclampsia-related vascular dysfunction [26]. These findings support early identification and individualized monitoring of twin pregnancies with PE to mitigate maternal risks, particularly postpartum hemorrhage. On the fetal side, the lack of significant differences in neonatal outcomes between singletons and twins at the same gestational age suggests that delivery timing remains the key factor to optimize neonatal prognosis. Future multicenter prospective studies are needed to validate these findings and optimize management strategies.

The main strength of this study lies in the extensive number of cases recruited for analysis, and the stratified analysis distinguishing between preterm and term preeclampsia. Additionally, the study benefited from detailed documentation of maternal complications and comprehensive neonatal management. As a single-center study, consistent antepartum and postpartum care across all cases ensured homogeneity in clinical management.

It is important to recognize the inherent limitations of this retrospective study. As it was conducted at a tertiary hospital with a neonatal intensive care unit, some preterm pregnancies were referred from primary care facilities, potentially introducing selection bias. Moreover, the publication of the ASPRE trial [19] in 2017, which advocated for aspirin use in high-risk pregnancies to prevent early-onset preeclampsia, may have led to a decline in preterm preeclampsia cases in subsequent years, further contributing to potential selection bias.

Twin pregnancies with preeclampsia exhibit a significantly higher rate of maternal complications, particularly postpartum hemorrhage and longer hospital stays. While the incidence of neonatal complications was similar between singletons and twins, preterm preeclampsia remains a major determinant of adverse neonatal outcomes, with low birthweight and delivery before 32 weeks being key predictors. Mode of delivery did not significantly influence maternal or neonatal complications.

## Data Availability

The data that support the findings of this study are not openly available due to reasons of sensitivity and are available from the corresponding author upon reasonable request.
